# Supplementary material for: Soluble Expression of Fc-Fused T Cell Receptors Allows Yielding Novel Bispecific T Cell Engagers
Source: Biomedicines. 2021 Jul 8;9(7):790. doi: 10.3390/biomedicines9070790 (PMC8301436; doi:10.3390/biomedicines9070790)
Supplement: Supplementary file 1 [file biomedicines-09-00790-s001.zip › biomedicines-1239618-supplementary.pdf]

Supplementary information

Soluble Expression of Fc−Fused T Cell Receptors Allows Yielding Novel Bispecific T Cell Engagers

Wen-Bin Zhao, Ying Shen, Wen-Hui Liu, Yi-Ming Li, Shi-Jie Jin, Ying-Chun Xu, Li-Qiang Pan, Zhan Zhou * and Shu-Qing Chen *

Institute of Drug Metabolism and Pharmaceutical Analysis & Zhejiang Provincial Key Laboratory of Anti-Cancer Drug Research, College of Pharmaceutical Sciences, Zhejiang University, Hangzhou 310058, China; pharmacy_zwb@zju.edu.cn (W.-B.Z.); shinesysy@foxmail.com (Y.S.); liuwenhui@zju.edu.cn (W.-H.L.); yiming_li@zju.edu.cn (Y.-M.L.); jinsj08@163.com (S.-J.J.); ycxu66@163.com (Y.-C.X.); panliqiang@zju.edu.cn (L.-Q.P.)

* Correspondence: zhanzhou@zju.edu.cn (Z.Z.); chenshuqing@zju.edu.cn (S.-Q.C.); Tel.: +86-0571-88208410 (Z.Z.); +86-0571-88208411 (S.-Q.C.)

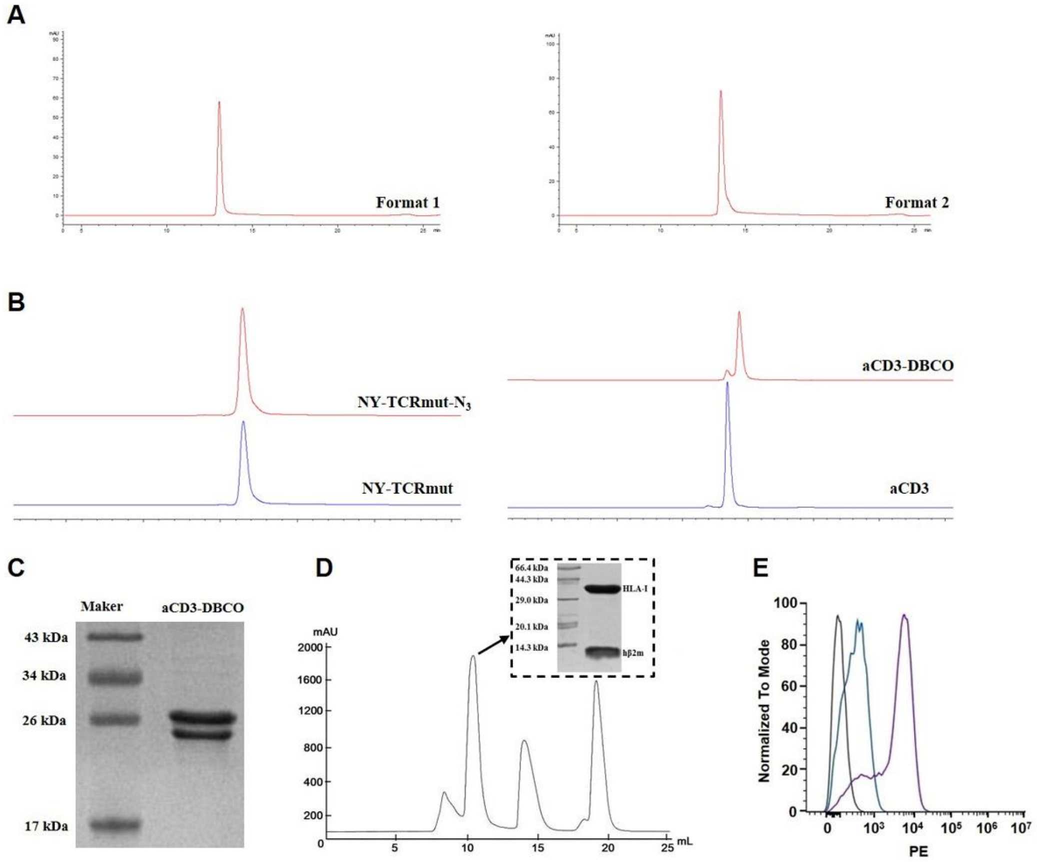

Figure S1. Analysis of the biologics used in this study. (A) RP-HPLC analysis of the two formats of NY-TCRmut. A single peak at about 13 min indicated high purity of NY-TCRmut. **(B)** RP-HPLC analysis of sortase A-mediated conjugation. The peak of aCD3-DBCO was shift to the right of aCD3, the peaks of NY-TCRmut and NY-TCRmut-N_3 were overlapped. The conjugation efficiency between GGG-PEG_4-DBCO and aCD3 was high, while the conjugation efficiency between GGG-PEG_3-N_3 and NY-TCRmut was difficult to confirm. **(C)** Analysis of the aCD3-DBCO by SDS-PAGE in denatured condition. aCD3-L (~23 kDa), aCD3-H (~25 kDa). **(D)** Purification and analysis of pHLA by molecular exclusion chromatography. The product of the 10-minute peak which contained HLA-I (~35 kDa) and β2m (~11 kDa), was the pHLA.

(E) NY-TCR/aCD3 simultaneously recognize CD3ε$^+$ and soluble NY/A02. Purple peak: NY-TCRmut/aCD3, green peak: NY-TCRwt/aCD3, brown peak: blank. The further to the right, the higher the MFI value.

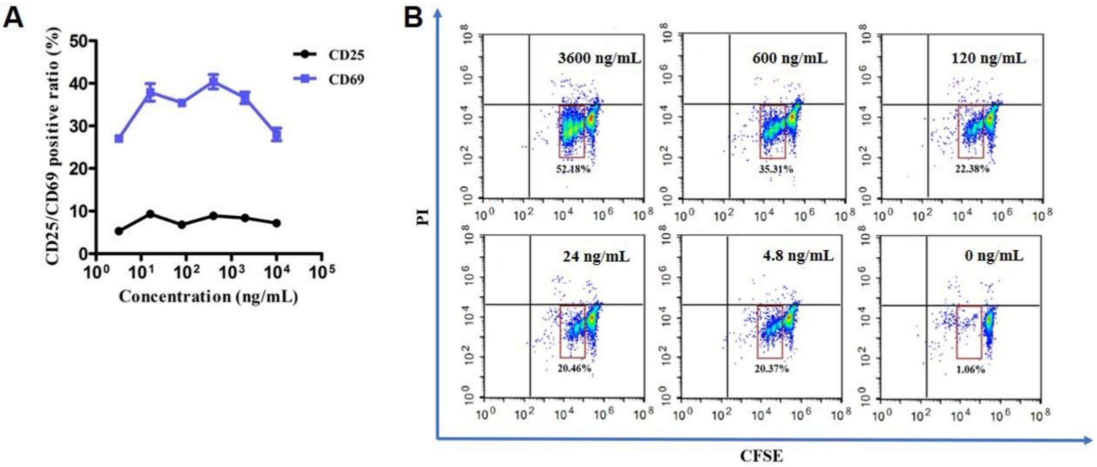

Figure S2. The T cell activation by NY-TCRmut/aCD3. (A) T cell activations were marked as CD69$^+$ or CD25$^+$ after treatment for 48 h (n=3). CD69$^+$ T cells were significantly increased, but CD25$^+$ T cells were slightly increased with different concentrations of NY-TCRmut/aCD3. **(B)** T cell proliferation after treatment for 72 h (n=1). T cells were pre-labelled with CFSE and the fluorescence intensity of CFSE was decreased with T cell proliferation. Cells in the red box were proliferating T cells.

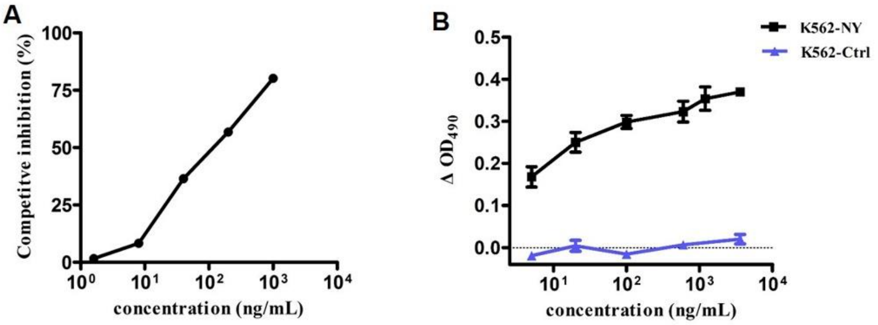

Figure S3. NY-TCRmut/aCD3 redirected ATC to specifically kill NY/A02$^+$ tumor cells. (A) Competitive inhibition of the NY-TCRmut/aCD3 activity by NY/A02. The lysis level was determined by cell apoptosis, and competition inhibition = apoptosis ratio of the test group/ apoptosis ratio of the control group (0 ng/mL NY/A02). **(B)** Lysis of K562-based tumor cells 24 h by ATC in the presence of titrated concentrations of NY-TCRmut/aCD3 (n=3). The lysis was determined by the LDH release assay.

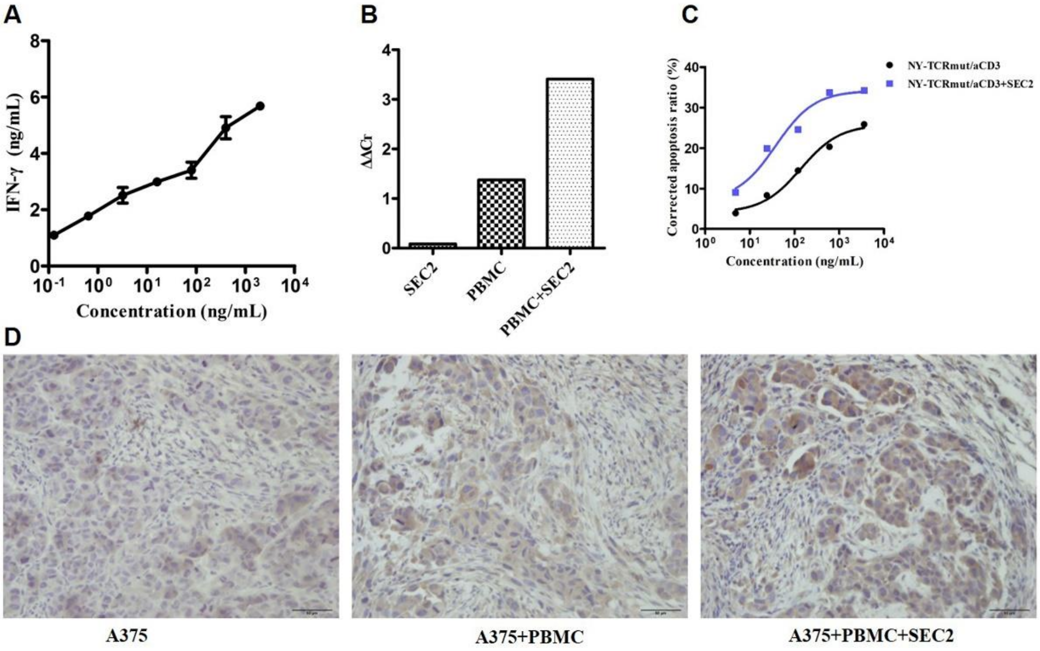

Figure S4. SEC2 enhanced the anti-tumor activity of NY-TCRmut/aCD3. (A) T cell activations by SEC2 were showed by IFN-γ assay (n=3). IFN-γ secretion was significantly increased in a dose-depended manner. **(B)** The HLA-A*02 expression level in A375 cells was up-regulated after stimulated by the secretory substances of SEC2-activated T cells. The HLA-A*02 expression level was determined by RT-PCR. **(C)** Lysis of K562-NY cells over 48 h by PBMC in the presence of titrated concentrations of NY-TCRmut/aCD3 with or without 2 ng/mL SEC2 (n=1). The lysis was determined by cell apoptosis. NY-TCRmut/aCD3 was able to redirect the lytic activity of T cells more effectively at different concentrations with 2 ng/mL SEC2. **(D)** SEC2 can promote the expression of pHLA *in vivo*. The expression level of pHLA in A375 xenograft models was determined by immunohistochemical staining with W6/32. The darker the brown, the higher the pHLA expression.

Table S1 Expression of NY-ESO-1/LAGE-$1_{157\text{-}165}$, HLA-A*02 and NY/A02 on different tumor cell lines

Cell lines	NY-ESO-1/LAGE-$1_{157\text{-}165}$	HLA-A*02	NY/ A02
K562	Negative	Negative	Negative
K562-Ctrl	Negative	Positive	Negative
K562-NY	Positive	Positive	4.67
A375	Positive	Positive	Not tested
A375-eGFP	Positive	Positive	Not tested
A375-NY	Positive	Positive	10.17

Table S2 Primers for real time PCR

Gene	Primer (5’ to 3’)	Tm (°C)
*HLA-A*0201*	TGGAGAACGGGAAGGAGACG (forward)<br>CAGTGTGATCTCCGCAGGGT (reverse)	55.5
GAPDH	TGGTGAAGACGCCAGTGGA (forward)<br>GCACCGTCAAGGCTGAGAAC (reverse)	50.0
NY-ESO-1/LAGE-1	GCTTGAGTTCTACCTCGCCA (forward)<br>AGCCAAAAACACGGGCAGAA (reverse)	60.0

Supplementary Methods and Results

Acquisition of tumor cell lines

The expression of HLA-A*02 of K562 and A375 cells was determined by flow cytometry using FITC labeled BB7.2 as the method mentioned in the HLA-A*02 expression assay. The total mRNA of K562 and A375 cells were extracted by Total RNA Miniprep Kit (Axygen Biosciences, Tewksbury, MA, USA). The mRNAs were transcribed into cDNAs using PrimeScript^{TM} RT Master Mix (Takara Bio, Tokyo, Japan), and then the cDNA was used to determine the expression of NY-ESO-1/LAGE-1 in K562 and A375 cells by PCR and sequencing. The K562-A2 cell line was constructed by stably transfecting HLA-A*0201 gene into K562 cells by our laboratory before. The gene of eGFP-ubiquitin-NY-ESO-1$_{157\text{-}165}$ and eGFP-ubiquitin-T790M were synthesized by the Sangon Biotech (Shanghai) Co., Ltd. (Shanghai, China), and then inserted into the pMH3 plasmid followed by stably transfected into K562-A2 cells to generate K562-NY and K562-Ctrl respectively. The eGFP-ubiquitin-NY-ESO-1$_{157\text{-}165}$ and eGFP genes were stably transfected into A375 cells to generate A375-NY and A375-eGFP respectively. The expression levels of NY/A02 in the cell lines were determined by flow cytometry using NY-TCRmut and PE labelled goat anti-human IgG Fc (ThermoFisher, Waltham, MA, USA) as the method mentioned in cell-binding assays. All the results here were showed in supplementary Table 1. Although A375 cells expressed HLA-A*0201 and NY-ESO-1/LAGE-1, the expression level of NY/A02 was too low to detect. Ubiquitin can mediate proteins into proteasome to degrade, so the expression of NY/A02 of A375-NY was significantly higher than that of A375.

RP-HPLC analysis

RP-HPLC was used for the sortase A reaction efficiency evaluation using a Varian PLRP-S 100 Å column (Tosoh Bioscience LLC, USA). The separation was performed with a mobile phase (Ultrapure water containing 0.1% TFA, acetonitrile) at the flow rate of 0.6 mL/min. As supplementary figure 1B showed, the peak of aCD3-DBCO was shifted to

the right of that of aCD3, resulting from the increased hydrophobic interaction between antibody and column resin. By comparing the peak area, we could deduce that the conjugation efficiency between GGG-PEG_4-DBCO and aCD3 was very high. However, the peaks of NY-TCRmut-N_3 and NY-TCRmut were overlapped, so we could not determine the conjugation efficiency between GGG-PEG_3-N_3 and NY-TCRmut.

Generation of pHLA

Genes of the ECD of HLA-A*0201 (tagged with Bir A recognition sequence for biotinylation) and human β2m were synthesized by the Sangon Biotech (Shanghai) Co., Ltd., and then inserted into the pET28a plasmid to construct expression plasmid. Then, ECDs of HLA-A*0201 and β2m were expressed as inclusion bodies in *Escherichia coli* followed by resolubilization. Peptides were synthesized by the Sangon Biotech (Shanghai) Co., Ltd. The pHLAs were refolded as the reported method and biotinylated using Bir A [1]. Finally, biotinylated pHLAs were purified using molecular exclusion chromatography (Superdex™ 75 Increase 10/300 GL, GE Healthcare Life Sciences, USA).

T cell proliferation assay

4×10^5 PBMC were labelled with CFSE (ThermoFisher, Waltham, MA, USA), and then were co-cultured with 1×10^5 A375 cells in 1 mL complete RPMI-1640 medium, containing different concentrations of NY-TCRmut/aCD3 at 37°C with 5% CO_2 and 95% humidity for 64 h in dark. After incubation, PBMC were collected and resuspended in ice-cold PBS, and then the fluorescence intensity of CFSE was analyzed by flow cytometry.

*HLA-A*02 expression assay by RT-PCR*

A375 cells were cultured in the 1 mL supernatant from PBMC or SEC2-activated T cells (donor1 or donor2), 1 mL complete RPMI-1640 medium and 1 mL complete RPMI-1640 medium containing 1 μg/mL SEC2-His respectively at 37°C with 5% CO_2 and 95% humidity for 48 h. After incubation, A375 cells were collected to extract mRNAs, and then the mRNAs were transcribed into cDNAs as the method mentioned above. The expression levels of HLA-A*02 and GAPDH (endogenous housekeeping gene) were measured by Real-time PCR on StepOne Real-time PCR system with SYBR Premix Ex Taq II Kit (Takara Bio, Tokyo, Japan). Primers used for Real-time PCR are listed in supplementary Table S2.

Activation of T cells by SEC2

3×10^4 A375 cells and 1.2×10^5 PBMC were co-cultured in 0.3 mL complete RPMI-1640 medium, containing different concentrations of SEC2 at 37°C with 5% CO_2 and 95% humidity for 48 h. After incubation, the supernatant was collected to

determine the concentration of IFN-γ by the IFN-γ human ELISA kit.

SEC2 up-regulate the expression level of pHLA-I in vivo

Two eight weeks old female Beige-SCID mice (Charles River Laboratories Co., Ltd., Beijing, China) were subcutaneously engrafted with A375 cells (2×10^6) and PBMC (4×10^6) and the other eight weeks old female Beige-SCID mouse was engrafted with A375 cells only (2×10^6) in the right armpit. The mice were treated with 200 ng/kg SEC2 or saline 1 h after engraftment. We then administrated 4 further doses every other day. The mice were euthanized the day after the last dose and tumor tissues were taken out for immunohistochemical staining with W6/32 (pHLA-I specific antibody, ThermoFisher, Waltham, MA, USA).

NY-TCR/aCD3 simultaneously recognize CD3ε and soluble NY/A02

Approximately 5×10^5 Jurkat cells were resuspended in ice-cold PBS and mixed with 10 μg/mL NY-TCR/aCD3. After 30 min incubation in 4°C, cells were washed and then incubated with 10 μg/mL NY/A02 for 30 min at 4°C. Cells were then washed, immunostained with PE labelled streptavidin (Sangon Biotech (Shanghai) Co., Ltd), and then analyzed by flow cytometry after washing with PBS.

Reference

1. Denkberg, G.; Cohen, C.J.; Segal, D.; Kirkin, A.F.; Reiter, Y. Recombinant human single-chain MHC-peptide complexes made from E. coli by in vitro refolding: Functional single-chain MHC-peptide complexes and tetramers with tumor associated antigens. *Eur. J. Immunol.* **2000**, *30*, 3522–3532.
